# Former SARS-CoV-2 Infection Was Related to Decreased VO_2_ Peak and Exercise Hypertension in Athletes

**DOI:** 10.3390/diagnostics13101792

**Published:** 2023-05-18

**Authors:** Karsten Keller, Oliver Friedrich, Julia Treiber, Anne Quermann, Birgit Friedmann-Bette

**Affiliations:** 1 Medical Clinic VII, Department of Sports Medicine, University Hospital Heidelberg, 69120 Heidelberg, Germany; 2 Department of Cardiology, University Medical Center of the Johannes Gutenberg-University Mainz, 55131 Mainz, Germany; 3 Center for Thrombosis and Hemostasis (CTH), University Medical Center of the Johannes Gutenberg-University Mainz, 55131 Mainz, Germany

**Keywords:** COVID-19, cardiopulmonary, exercise hypertension, exercise testing, spiroergometry

## Abstract

The impact of former COVID-19 infection on the performance of athletes is not fully understood. We aimed to identify differences in athletes with and without former COVID-19 infections. Competitive athletes who presented for preparticipation screening between April 2020 and October 2021 were included in this study, stratified for former COVID-19 infection, and compared. Overall, 1200 athletes (mean age 21.9 ± 11.6 years; 34.3% females) were included in this study from April 2020 to October 2021. Among these, 158 (13.1%) athletes previously had COVID-19 infection. Athletes with COVID-19 infection were older (23.4 ± 7.1 vs. 21.7 ± 12.1 years, *p* < 0.001) and more often of male sex (87.7% vs. 64.0%, *p* < 0.001). While systolic/diastolic blood pressure at rest was comparable between both groups, maximum systolic (190.0 [170.0/210.0] vs. 180.0 [160.0/205.0] mmHg, *p* = 0.007) and diastolic blood pressure (70.0 [65.0/75.0] vs. 70.0 [60.0/75.0] mmHg, *p* = 0.012) during the exercise test and frequency of exercise hypertension (54.2% vs. 37.8%, *p* < 0.001) were higher in athletes with COVID-19 infection. While former COVID-19 infection was not independently associated with higher blood pressure at rest and maximum blood pressure during exercise, former COVID-19 infection was related to exercise hypertension (OR 2.13 [95%CI 1.39–3.28], *p* < 0.001). VO_2_ peak was lower in athletes with compared to those without COVID-19 infection (43.4 [38.3/48.0] vs. 45.3 [39.1/50.6] mL/min/kg, *p* = 0.010). SARS-CoV-2 infection affected VO_2_ peak negatively (OR 0.94 [95%CI 0.91–0.97], *p* < 0.0019). In conclusion, former COVID-19 infection in athletes was accompanied by a higher frequency of exercise hypertension and reduced VO_2_ peak.

## 1. Introduction

In December 2019, the first pneumonia and respiratory illness cases of unknown origin were detected in China. The causative pathogen was identified and announced by the Department of Zoonoses at the National Institute of Communicable Disease Control and Prevention (Chinese Center for Disease Control and Prevention) as severe acute respiratory syndrome coronavirus 2 (SARS-CoV-2) [1,2,3]. On 30 January 2020, the World Health Organization (WHO) declared this outbreak of coronavirus disease 2019 (COVID-19) a public health emergency of international concern [3].

The COVID-19 pandemic has brought into sharp focus the weaknesses of healthcare systems and the shared frailty of societies in the face of common threats [4,5]. Global health faces a sovereignty paradox as health is mostly a national responsibility, whereas a pandemic does not stop at borders and must be managed as a global challenge [4,6]. The first cases of COVID-19 infection in Germany were detected at the end of January 2020 with a fast and strong spread in the German population [7,8,9].

The COVID-19 pandemic dramatically changed lifestyles worldwide, including sports activities [10,11]. Initially, many limitations were introduced to contain the spread of the pandemic, such as canceled competitive events, limited access to training facilities, and sanitary restrictions [10,11]. After the first lockdown measures, the restrictions were revoked step by step, beginning with professional sports such as soccer, followed by other sports in Germany.

Already early in the pandemic, reports regarding myocardial injury among patients hospitalized with COVID-19 raised concern that athletes may also face this risk for cardiac involvement [11,12]. At the time, the prevalence of clinical myocarditis, myocardial involvement, and myocardial injury in athletes was widely unclear [12]. Later, conducted studies revealed a low prevalence of cardiac involvement and a low risk of clinical events during and after COVID-19 infection in athletes [13,14].

Although most competitive athletes have an asymptomatic or mildly symptomatic SARS-CoV-2 infection course and are a low-risk population to develop cardiac and other complications after SARS-CoV-2 infection [8,14], a post-COVID-19 screening strategy primarily based on symptoms should detect cardiac involvement and other complications from SARS-CoV-2 infection and should enable the safe and early return to sports of affected athletes after infection [11,12,14,15,16].

However, the incidence, complications, and performance consequences of COVID-19 infection in athletes are not fully understood [11,14]. A few studies suggest lower performance level capacities despite being cured of COVID-19 infection, with decreased VO_2_ peak values in individuals with former COVID-19 infection [17,18]. Thus, the main aim of the present study was to identify differences in athletes with former COVID-19 infections in comparison to those athletes without COVID-19 infection presenting for their preparticipation screening examination. The hypothesis of this present study was that the performance of competitive athletes is significantly influenced by former COVID-19 infection.

## 2. Methods

### 2.1. Study Design

**Figure 1:**
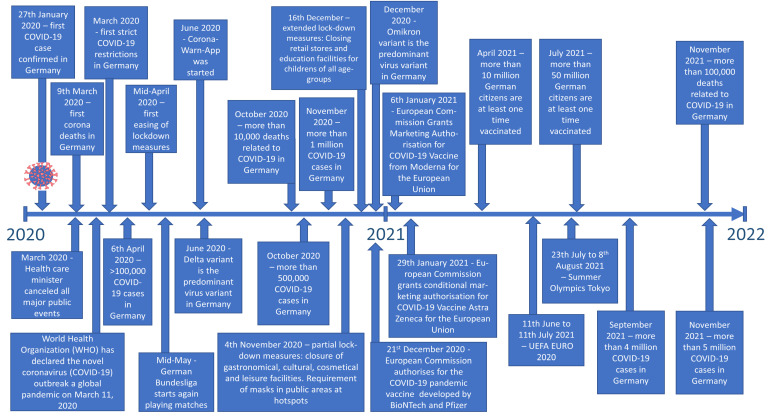
Timeline of COVID-19 pandemic in Germany.

We performed a retrospective analysis of standard data of athletes of any age who presented for their preparticipation screening examination at the Department of Sports Medicine (Medical Clinic VII) of the University Hospital Heidelberg (Germany) between April 2020 and October 2021. For a better understanding of the impact of lockdown measures and time trends regarding the COVID-19 pandemic in Germany, see Figure 1. The study was primarily designed to evaluate the blood pressure adaptations and exercise hypertension in athletes and was already described in detail in one previously published paper [19].

### 2.2. Study Sample and Enrolled Subjects

**Figure 2:**
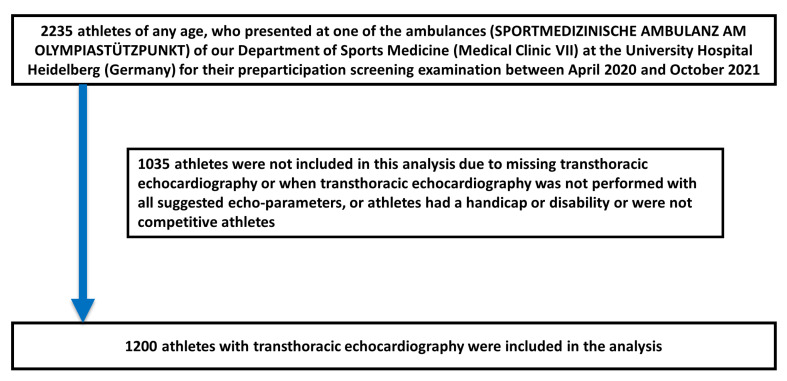
Flowchart of the study.

Athletes were eligible for inclusion in this study if they performed regular training for competition and presented for preparticipation screening examination or post-COVID-19 return-to-sport testing [12,16,19,20,21]. Athletes without transthoracic echocardiography, or for whom echocardiography was performed, but not all suggested echo-parameters in the echocardiography were available, or athletes who had a handicap or disability or were not competitive athletes were not included in the study (see flowchart in Figure 2).

### 2.3. Instruments/Procedures

The impact of former COVID-19 infection on blood pressure and exercise outcomes such as VO_2_ peak were investigated. The transthoracic echocardiography was performed with the Vivid S6 device (General Electric, Boston, MA, USA). The exercise testing was performed either on two different bicycle devices (Excalibur Sport, Lode, Groningen, The Netherlands) or two different treadmill devices (PPS-70-MED WEISS or ELG, WOODWAY GmbH, European headquarters: Weil am Rhein, Germany). The device Geratherm (Ergostik) of the firm AMEDTEC Medizintechnik Aue GmbH (Aue-Bad Schlema, Germany) was used for the spiroergometric testing.

### 2.4. Ethical Aspects

The requirement for informed consent for this study was waived as we used only anonymized retrospective data routinely collected during the health screening process. Studies in Germany involving a retrospective analysis of diagnostic standard data of anonymized patients do not require an ethics statement, in accordance with German law.

### 2.5. Definitions and Assessed Parameters

For all athletes included and investigated in this study, transthoracic echocardiography was performed. Echocardiographic parameters were defined according to current guidelines [21,22].

As previously described [19], left ventricular hypertrophy (LVH) was defined as (I) septal or posterior left ventricular (LV) wall diameter ≥13 mm [21,23] or (II) LV mass >162 g in females or >224 g in males [22]. LV mass was computed according to the established 2D echocardiography area–length method: LV mass = 0.80 * (1.04 * [(septal LV wall thickness + LV end-diastolic diameter + posterior LV wall thickness)^3^ − (LV end-diastolic diameter)^3^]) + 0.6 g [22]. LVH was present if one or both of the definitions I or II was applicable [19].

Exercise hypertension was defined on the basis of the systolic blood pressure (BP)/MET slope method [24,25,26,27]: Δ regarding systolic BP was calculated as maximum systolic BP during exercise − systolic BP at rest and was indexed by the increase in MET from rest (Δ regarding MET was calculated as peak MET − 1) to obtain the systolic BP/MET slope [26]. In accordance with previous studies, a cut-off value >6.2 mmHg/MET was used to define exercise hypertension [19,24,26]. The MET value was calculated based on the athletes’ VO_2_ peak value during exercise testing, as recommended by the ACSM guidelines (MET = VO_2_ peak/3.5 mL × kg^−1^ × min^−1^) [28].

Exercise testing was performed according to current guidelines with an electrocardiogram (ECG) and BP measurements at the end of every load level. The exercise test was stopped if the athlete was at his or her maximum capacity, or if stopping criteria according to current guidelines forced the exercise test to stop [20,21].

Obesity was defined as body mass index (BMI) ≥30 kg/m^2^ according to the World Health Organization (WHO) [19,29].

### 2.6. Statistics

The included athletes of this present study were stratified for the presence of former COVID-19 infection (confirmed by laboratory testing), and athletes with and without former COVID-19 infection were compared with the help of the Wilcoxon–Mann–Whitney U-test for continuous variables and Fisher’s exact test or chi^2^ test for categorical variables, as appropriate. Data of continuous variables were presented as median and interquartile ranges and categorical variables as absolute numbers with related percentages.

We performed univariate and multivariate logistic regressions to investigate the association between different assessed parameters on one hand and the presence of former COVID-19 infection on the other hand. This approach was selected to investigate the influence of former COVID-19 infection on the different parameters. Multivariate logistic regression models were adjusted for age, sex, and BMI in order to prove the independence of these statistical results regarding these known influencing parameters. Results of the logistic regressions are presented as odds ratio (OR) and 95% confidence interval (CI).

All of the statistical analyses were carried out with the use of SPSS software (IBM Corp. Released 2017. IBM SPSS Statistics for Windows, Version 25.0. Armonk, NY, USA). Only *p* values < 0.05 (two-sided) were considered to be statistically significant. No adjustment for multiple testing was applied in the present analysis.

## 3. Results

Overall, 1200 athletes (mean age 21.9 ± 11.6 years; 411 [34.3%] females) were included in the present study between April 2020 and October 2021. Most included athletes were in the second and third decade of life. In total, 73.3% were leading athletes at a regional or national level. The mean training duration of the athletes was 8 years. Among the included patients, 1.8% were obese and 3.4% reported nicotine abuse (Table 1). In total, 158 (13.1%) athletes previously had COVID-19 infection.

### 3.1. Comparison of Athletes with and without Former COVID-19 Infection

#### 3.1.1. Patient Characteristics

Athletes with former COVID-19 infection were slightly older (23.4 ± 7.1 vs. 21.7 ± 12.1 years, *p* < 0.001), more often of male sex (87.7% vs. 64.0%, *p* < 0.001) and had higher BMI (23.5 [21.9/25.6] vs. 22.3 [20.4/24.3] kg/m^2^, *p* < 0.001) in light of lower body fat (10.5 [8.5/15.1] vs. 11.8 [8.7/16.8], *p* = 0.021) in comparison to athletes without COVID-19 infection (Table 1). The frequencies of the investigated cardiovascular risk factors obesity and nicotine abuse were similar between both groups.

#### 3.1.2. Echocardiographic and Blood Pressure

While systolic and diastolic blood pressure values at rest were comparable between athletes with and without former COVID-19 infection, maximum systolic (190.0 [170.0/210.0] vs. 180.0 [160.0/205.0] mmHg, *p* = 0.007) and diastolic blood pressure (70.0 [65.0/75.0] vs. 70.0 [60.0/75.0] mmHg, *p* = 0.012) during the exercise test were both higher in athletes with COVID-19 infection compared to those without. Notably, exercise hypertension was more often observed in athletes with former COVID-19 infection (54.2% vs. 37.8% with an increase in blood pressure >6.2 mmHg/MET, *p* < 0.001) than without, whereby left ventricular hypertrophy (29.9% vs. 20.3%, *p* = 0.006) was also more prevalent. Median values of left ventricular mass (194.4 [157.9/226.4] vs. 164.5 [132.8/200.8] g, *p* < 0.001) in combination with calculated absolute heart volume (934.4 [769.5/1034.0] vs. 772.3 [642.0/912.2] mL, *p* < 0.001) were higher in athletes with former COVID-19 infection (Table 1).

While both atria were larger in athletes with former COVID-19 infection, tricuspid annular plane systolic excursion (TAPSE) was higher in athletes with compared to those without former COVID-19 infection (2.60 [2.22/2.90] vs. 2.50 [2.20/2.80] cm, *p* = 0.003) (Table 1).

### 3.2. Risk Factors for COVID-19 Infection

Male sex (OR 1.60 [95%CI 1.06–2.40], *p* = 0.026) and higher BMI (OR 1.13 [95%CI 1.07–1.19], *p* < 0.001) were statistically independent risk factors for COVID-19 infection, whereas age, obesity, and nicotine abuse were not independently associated with COVID-19 infection (Table 2).

### 3.3. Impact of SARS-CoV-2 Infection on Blood Pressure

**Figure 3:**
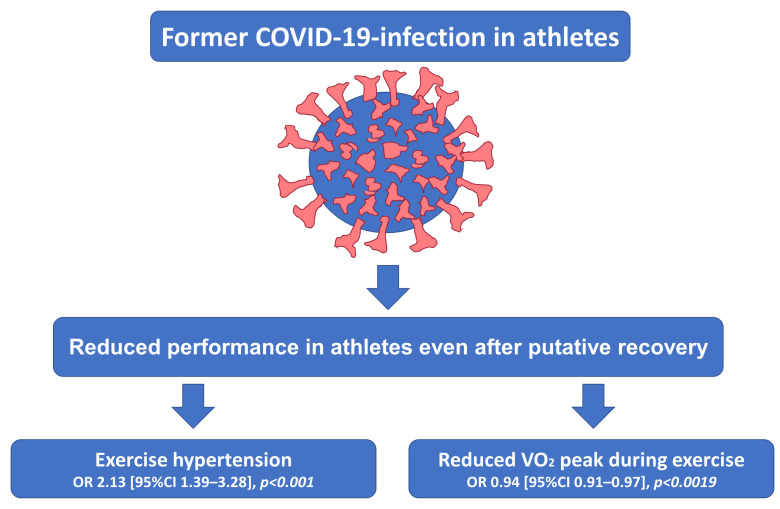
Impact of former SARS-CoV-2 infection on exercise parameters in athletes.

While former COVID-19 infection was not independently associated with higher blood pressure values at rest and higher maximum blood pressure values during exercise, former COVID-19 infection was related to exercise hypertension (OR 2.13 [95%CI 1.39–3.28], *p* < 0.001) (Table 2, Figure 3).

### 3.4. Impact of SARS-CoV-2 Infection on Exercise Parameters

VO_2_ peak value during exercise was lower in athletes with former COVID-19 infection in comparison to those athletes without infection (43.4 [38.3/48.0] vs. 45.3 [39.1/50.6] mL/min/kg, *p* = 0.010). SARS-CoV-2 infection negatively affected VO_2_ peak values during exercise (OR 0.94 [95%CI 0.91–0.97], *p* < 0.0019) in light of an uninfluenced respiratory exchange ratio (RER) and maximum lactate value during exercise testing (Table 2, Figure 3).

### 3.5. Impact of SARS-CoV-2 Infection on Echocardiographic Parameters

Former COVID-19 infection was independently associated with a larger left ventricular end-diastolic diameter (OR 1.09 [95%CI 1.04–1.14], *p* < 0.001), larger left atrial area (OR 1.14 [95%CI 1.08–1.21], *p* < 0.001), larger right atrial area (OR 1.08 [95%CI 1.02–1.14], *p* = 0.010), and decreased E/E’ quotient (OR 0.83 [95%CI 0.73–0.95], *p* = 0.008 (Table 2).

### 3.6. Symptoms during the SARS-CoV-2 Infection of Athletes with Former COVID-19 Infection

**Figure 4:**
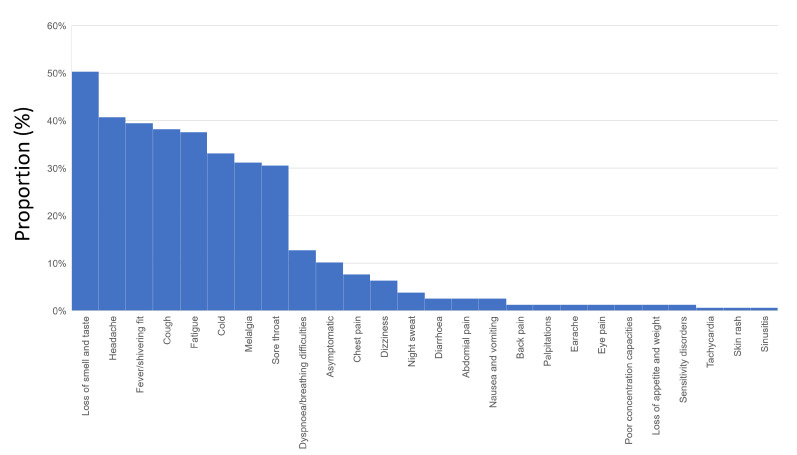
Symptoms during COVID-19 infection.

Overall, 10.2% of the SARS-CoV-2 infections were asymptomatic. The most prevalent symptoms were loss of smell and/or taste, headache, fever, and cough (Figure 4). Dyspnea/breathing difficulties occurred in 12.7% and chest pain in 7.6% of the infections.

**Figure 5:**
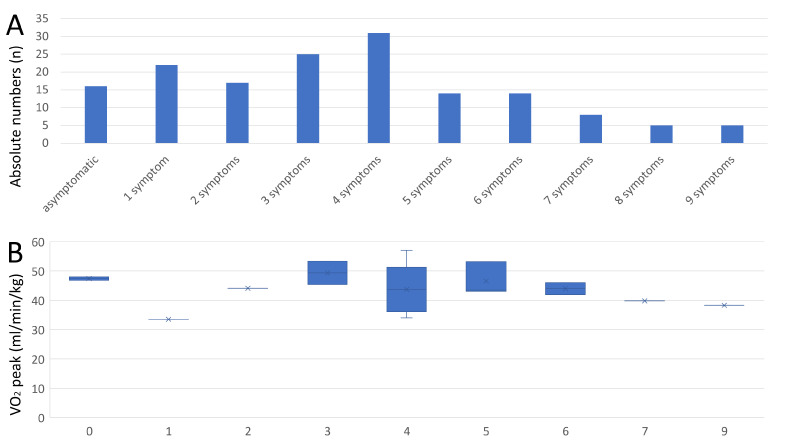
Total numbers of symptoms during COVID-19 infection and peak VO_2_ value. **Panel A**—Total number of athletes stratified by total number of symptoms during COVID-19 infection. **Panel B**—Peak VO_2_ value of athletes stratified by total number of symptoms during COVID-19 infection.

The athletes with former COVID-19 infection most often reported three or four symptoms during infection (Figure 5A). Remarkably, increasing numbers of symptoms during COVID-19 infection were not related to decreased VO_2_ peak values (β −0.16 [95%CI −1.85 to 0.97], *p* = 0.520) (Figure 5B). An increasing number of symptoms were also not independently associated with exercise hypertension (OR 0.45 [95%CI 0.19–1.07], *p* = 0.071).

**Figure 6:**
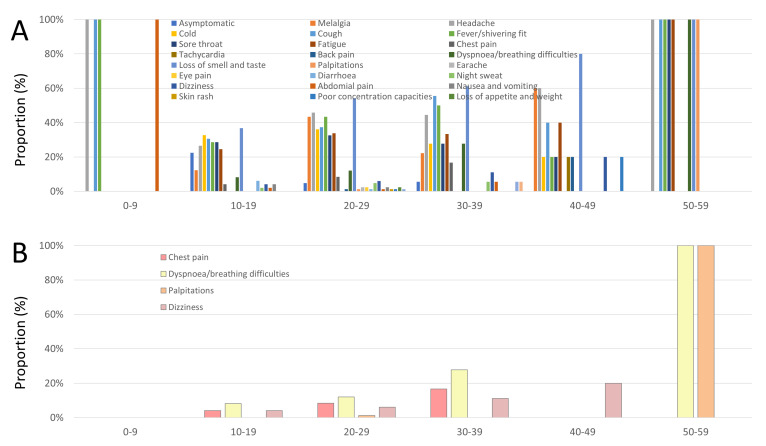
Symptoms during COVID-19 infection stratified by age decades. **Panel A**—Symptoms during COVID-19 infection stratified by age decades. **Panel B**—Chest pain, dyspnea, palpitations, and dizziness during COVID-19 infection stratified by age decades.

Figure 6A illustrates the proportions of symptoms in athletes with former COVID-19 infection in different age decades of life. Interestingly, the proportion of athletes with breathing difficulties/dyspnea and chest pain increased with rising age (Figure 6B). Breathing difficulties/dyspnea (OR 0.90 [95%CI 0.76–1.07], *p* = 0.232) and chest pain (OR 0.93 [95%CI 0.78–1.12], *p* = 0.434) were not independently associated with decreased VO_2_ peak values.

## 4. Discussion

The COVID-19 pandemic constitutes an unforeseen infectious disease burden and has had unprecedented impacts on healthcare systems worldwide [30]. Lifestyles changed dramatically during the pandemic, including sports activities in elite sports and sports for the general public [10]. Especially during the initial period of the pandemic, many limitations were introduced to prevent the further spread of SARS-CoV-2 infections, such as canceled competitive events, limited access to training facilities, and sanitary restrictions [8,10,29,31].

At the beginning of the pandemic, many people expected that highly trained athletes would be strong and healthy enough to fend off a SARS-CoV-2 infection. However, already in March 2020, it became clear that strong and healthy athletes also got infected and more than a hundred athletes were identified to be affected by the SARS-CoV-2 [31,32].

Although in most young athletes, an asymptomatic or mildly symptomatic SARS-CoV-2 infection course has been observed, and these patients have to be considered a low-risk population for developing cardiac and other complications after SARS-CoV-2 infection [8,14], a post-COVID-19 screening strategy to enable a safe and early return to sports primarily based on symptoms was implemented in the German sports system to face the fear regarding the development of cardiac involvement and other complications from SARS-CoV-2 infection [11,12,14,15,16,31].

In our large study with 1200 athletes who presented at the Department of Sports Medicine (Medical Clinic VII) of the University Hospital Heidelberg (Germany) for their preparticipation screening examination between April 2020 and October 2021, we observed a proportion of 13.1% of athletes with former COVID-19 infection.

In contrast to the results of a large nationwide epidemiological study demonstrating that the total number of female COVID-19 patients exceeds the total number of male COVID-19 patients in the first four decades of life [33], our data revealed that the majority of athletes with former COVID-19 infection were of male sex. In this context, it has to be mentioned that male sex was in several studies identified as a risk factor for adverse outcomes in patients with COVID-19 infection [8,29,34,35,36].

While obesity was also identified in studies as a risk factor for adverse events during COVID-19 infection [8,29,37,38], data regarding the impact of smoking on the disease course were inconsistent [39,40]. Our study demonstrated similar frequencies of obesity and nicotine abuse in athletes with and without former COVID-19 infection. In this context, male sex and higher BMI were independently related to COVID-19 infection, whereas age, obesity, and nicotine abuse were not.

Regarding blood pressure and arterial hypertension, studies observed a rise in blood pressure among adults in the United States of America during the COVID-19 pandemic [41,42]. Although we could not detect an increase in systolic and diastolic blood pressure levels at rest in athletes with former COVID-19 infection, maximum systolic and diastolic blood pressure during the exercise test were both higher in athletes with former COVID-19 infection compared to those without. In addition, exercise hypertension was more often present in athletes with former COVID-19 infection. Among these results of our study, the multivariate regressions confirmed that former COVID-19 infection was not independently associated with higher blood pressure values at rest and demonstrated that higher maximum blood pressure values during exercise were also not independently related to former COVID-19 infection. In contrast, the risk for exercise hypertension was 2.1-fold higher in athletes with former COVID-19 infection in comparison to those without former COVID-19 infection independently of age, sex, and BMI. This is an important finding since exercise hypertension in athletes might be one sign of persisting exercise intolerance [13,43,44]. Besides these findings and in accordance with the study of Singh et al. [44], we detected a significantly lower VO_2_ peak in athletes with former COVID-19 infection. The VO_2_ peak was 4% lower than in those athletes without former infection. This finding was confirmed by the multivariate regression model, revealing a 0.94-fold decreased VO_2_ peak in athletes with former COVID-19 infection independently of age, sex, and BMI. This finding is in accordance with two previously published study results, demonstrating also lower VO_2_ peak values after former COVID-19 infection [17,18]. It has been reported that exercise limitation is common and a frequent manifestation of post-COVID-19 syndrome in the first months after mild or moderate COVID-19 infection [44]. It has been shown that peripheral, rather than central cardiac limitation to exercise, based on diffusion defect in oxygen delivery contributes primarily decrease aerobic exercise capacity [44]. In addition, exaggerated hyperventilatory response and dysfunctional breathing during exercise affect aerobic exercise capacity [44,45,46]. Thus, the results of our study and evidence from the literature lead to the assumption that post-COVID-19 return-to-sport testing before resuming competitive sports might be helpful to identify athletes who may benefit from careful increases in training intensity after COVID-19 infection.

As expected and in accordance with the mentioned recommendations, previously published studies revealed a low prevalence of cardiac involvement and a low risk of clinical events during and after COVID-19 infection in athletes [13,14]. However, in a large, multi-center study, cardiac abnormalities were observed in half of all COVID-19 patients undergoing echocardiography [47]. Since in the mentioned study, the echocardiography was performed during acute COVID-19 infection [47], our study results revealed only some smaller deviations in the group of athletes with former COVID-19 infection. Since larger atrial and left-ventricular diameters in athletes with former COVID-19 infection might be attributed to older age and therefore longer sport activity periods, a lower (but still within the normal range) E/E′ quotient might be the result of other causes and might be influenced by former COVID-19 infection.

As previously reported by others [48,49], the symptoms most frequently reported by athletes with former COVID-19 infection were loss of smell and/or taste, headache, fever, and cough. In our study, approximately 10% of the SARS-CoV-2 infections were asymptomatic, which is lower than in the literature [48,49], but is of course highly influenced by the test strategy. While dyspnea/breathing difficulties occurred more commonly in the athletes included in our study, the proportion of athletes with chest pain during and after the COVID-19 infection was similar to results of previously published studies [48]. Remarkably, the proportion of athletes with breathing difficulties/dyspnea and chest pain increased with rising age, whereas breathing difficulties/dyspnea and chest pain were not independently associated with decreased VO_2_ peak values.

Risk and promotion factors of complications during and after COVID-19 infection, as well as those of performance reduction in athletes after COVID-19 infection, are incompletely understood [14]. Our study contributes to the evidence that former COVID-19 infection might affect the performance of athletes even after putative recovery after COVID-19 infection. Although the observed deviations were small, in highly trained athletes, these small deviations may and could be crucial to whether the athletes could maintain their performance level and might be decisive for winning or losing in competitive sports.

## 5. Limitations

There are certain limitations of our study that require consideration. First, the study is a retrospective monocenter study. Second, we used diagnostic standard data of anonymized patients and therefore cannot provide follow-up data of these patients. Third, for better comparison, athletes for whom transthoracic echocardiography was not performed were not included in the study. Fourth, since we included athletes of all ages, age might have influenced the results.

## 6. Conclusions

Former COVID-19 infection in athletes was accompanied by reduced VO_2_ peak during exercise testing and influenced blood pressure response during exercise with a higher frequency of exercise hypertension. Thus, former COVID-19 infection might affect the performance of athletes even after putative recovery after COVID-19 infection. The results of our study lead to the assumption that post-COVID-19 return-to-sport testing before resuming competitive sports might be helpful to identify athletes who may benefit from careful increases in training intensity.

## Figures and Tables

**Table 1:** Characteristics of the 1200 examined athletes stratified for SARS-CoV-2 infection.

Parameters	Athletes without SARS-CoV-2 Infection (*n* = 1043; 86.9%)	Athletes with SARS-CoV-2 Infection (*n* = 157; 13.1%)	*p*-Value
**Age (in years)**	21.7 ± 12.1	23.4 ± 7.1	<0.001
**Female sex**	376 (36.0%)	35 (22.3%)	<0.001
**Body height (cm)**	175.0 (167.0/181.0)	181.0 (172.8/187.0)	<0.001
**Body weight (kg)**	68.7 (59.0/78.5)	79.0 (67.6/87.1)	<0.001
**Body mass index (kg/m^2^)**	22.3 (20.4/24.3)	23.5 (21.9/25.6)	<0.001
**Body fat (%)**	11.8 (8.7/16.8)	10.5 (8.5/15.1)	0.021
**Leading athletes at a regional and national level**	766 (73.4%)	113 (72.0%)	0.669
Cardiovascular risk factors			
**Nicotine abuse**	34 (3.3%)	7 (4.5%)	0.441
**Obesity**	16 (1.5%)	6 (3.8%)	0.057
Blood pressure values			
**Systolic blood pressure at rest (mmHg)**	115.0 (110.0/120.0)	120.0 (110.0/125.0)	0.640
**Diastolic blood pressure at rest (mmHg)**	70.0 (60.0/75.0)	70.0 (65.0/75.0)	0.360
**Maximum systolic blood pressure during exercise (mmHg)**	180.0 (160.0/205.0)	190.0 (170.0/210.0)	0.007
**Maximum diastolic blood pressure during exercise (mmHg)**	75.0 (70.0/80.0)	75.0 (70.0/80.0)	0.012
**Exercise hypertension (defined by systolic blood pressure (BP)/MET slope method with cut-off value >6.2 mmHg/MET)**	199 (37.8%)	77 (54.2%)	<0.001
**Exercise parameters**			
**VO_2_ peak (mL/min/kg)**	45.3 (39.1/50.6)	43.4 (38.3/48.0)	0.010
**Maximal respiratory exchange** **ratio (RER)**	1.15 (1.11/1.20)	1.15 (1.10/1.21)	0.807
**Maximum lactate value**	9.42 (7.81/11.17)	9.52 (7.54/11.29)	0.826
**Echocardiographic parameters**			
**Left ventricular hypertrophy**	212 (20.3%)	47 (29.9%)	0.006
**Left ventricular mass (g)**	164.5 (132.8/200.8)	194.4 (157.9/226.4)	<0.001
**Aortic valve regurgitation**	79 (7.6%)	7 (4.5%)	0.158
**Mitral valve regurgitation**	565 (54.2%)	102 (65.0%)	0.011
**Tricuspid valve regurgitation**	153 (14.7%)	16 (10.2%)	0.310
**Pulmonary valve regurgitation**	109 (10.5%)	6 (3.8%)	0.006
**Heart volume in total (mL)**	772.3 (642.0/912.2)	934.4 (769.5/1034.0)	<0.001
**Heart volume related to body weight (mL/kg)**	11.4 (10.2/12.5)	11.7 (10.9/12.4)	0.015
**Left ventricular ejection fraction measured by Simpson method (%)**	65.0 (62.0/69.0)	65.0 (63.0/68.0)	0.778
**Left ventricular end-diastolic diameter (cm)**	49.0 (46.0/53.0)	52.0 (49.0/55.0)	<0.001
**Left atrial area (cm^2^)**	13.7 (11.4/15.7)	15.1 (13.3/17.5)	<0.001
**Right atrial area (cm^2^)**	13.5 (11.2/15.8)	15.1 (12.7/17.0)	<0.001
**Tricuspid annular plane systolic excursion (TAPSE, cm)**	2.50 (2.20/2.80)	2.60 (2.22/2.90)	0.003
**Systolic pulmonary artery pulmonary pressure (mmHg)**	20.0 (17.0/23.4)	20.2 (17.0/22.0)	0.651
**E/A quotient**	2.6 (1.9/3.6)	2.5 (1.8/3.6)	0.497
**E/E’ quotient**	4.8 (4.1/5.7)	4.5 (3.7/5.5)	0.007

**Table 2:** Association between SARS-CoV-2 infection and different measures (univariate and multivariate logistic regression model).

	SARS-CoV-2 Infection			
	Univariate Regression Model		Multivariate Regression Model (Adjusted for Age, Sex, and BMI)	
	OR (95% CI)	*p*-Value	OR (95% CI)	*p*-Value
**Age (in years)**	1.01 (1.00–1.03)	0.100	1.00 (0.98–1.01)	0.749
**Male sex**	1.97 (1.32–2.92)	<0.001	1.60 (1.06–2.40)	0.026
**Body mass index (kg/m^2^)**	1.14 (1.09–1.20)	<0.001	1.13 (1.07–1.19)	<0.001
**Body fat (%)**	0.95 (0.91–0.99)	0.007	0.90 (0.84–0.97)	0.003
**Cardiovascular risk factors**				
**Nicotine abuse**	1.39 (0.60–3.18)	0.443	1.09 (0.45–2.65)	0.844
**Obesity**	2.55 (0.98–6.62)	0.054	1.97 (0.74–5.21)	0.173
**Blood pressure values**				
**Systolic blood pressure at rest (mmHg)**	1.00 (0.97–1.01)	0.979	0.98 (0.96–1.00)	0.009
**Diastolic blood pressure at rest (mmHg)**	1.01 (0.99–1.03)	0.568	0.99 (0.97–1.01)	0.260
**Maximum systolic blood pressure during exercise (mmHg)**	1.007 (1.001–1.014)	0.017	0.998 (0.991–1.006)	0.685
**Maximum diastolic blood pressure during exercise (mmHg)**	1.024 (1.006–1.043)	0.010	1.013 (0.992–1.033)	0.220
**Exercise hypertension (defined by systolic blood pressure (BP)/MET slope method with cut-off value >6.2 mmHg/MET)**	1.96 (1.34–2.84)	<0.001	2.13 (1.39–3.28)	<0.001
**Exercise parameters**				
**VO_2_ peak**	0.974 (0.952–0.997)	0.029	0.943 (0.913–0.973)	<0.001
**Maximal respiratory exchange** **ratio (RER)**	0.80 (0.06–10.04)	0.864	0.72 (0.05–9.60)	0.801
**Maximum lactate value**	1.02 (0.91–1.15)	0.710	1.00 (0.89–1.13)	0.948
**Echocardiographic parameters**				
**Left ventricular hypertrophy**	1.68 (1.15–2.43)	0.007	1.28 (0.85–1.94)	0.236
**Left ventricular mass**	1.010 (1.006–1.013)	<0.001	1.005 (1.000–1.010)	0.054
**Left ventricular ejection fraction measured by Simpson method (%)**	1.00 (0.97–1.04)	0.817	1.02 (0.98–1.06)	0.431
**Left ventricular end-diastolic diameter (cm)**	1.12 (1.08–1.17)	<0.001	1.09 (1.04–1.14)	<0.001
**Left atrial area (cm^2^)**	1.18 (1.11–1.24)	<0.001	1.14 (1.08–1.21)	<0.001
**Right atrial area (cm^2^)**	1.12 (1.06–1.17)	<0.001	1.08 (1.02–1.14)	0.010
**Tricuspid annular plane systolic excursion (TAPSE, cm)**	1.98 (1.33–2.96)	<0.001	1.44 (0.94–2.21)	0.097
**Systolic pulmonary artery pulmonary pressure (mmHg)**	0.99 (0.94–1.04)	0.649	0.98 (0.93–1.03)	0.430
**E/A quotient**	0.94 (0.81–1.08)	0.358	0.98 (0.85–1.13)	0.781
**E/E’ quotient**	0.82 (0.72–0.94)	0.004	0.83 (0.73–0.95)	0.008

## Data Availability

The data presented in this study are available upon request from the corresponding author.
